# Osteoporosis treatment with biosimilars in Malaysia and Thailand: a budget impact analysis

**DOI:** 10.1007/s11657-026-01752-3

**Published:** 2026-08-13

**Authors:** Linda Siachalinga, Arun Jones, Kyoo Kim, Martin Downes, Igor Solev, Kenneth Lee, Thanut Valleenukul, Hansoo Kim

**Affiliations:** 1 Health Economics Group, School of Medicine and Dentistry, Griffith University, Gold Coast, 58 Parklands Drive, Southport, QLD 4215 Australia; 2 Abbott Product Operations AG, Allschwil, Switzerland; 3 School of Medicine and Health Sciences, Monash University Malaysia, Bandar Sunway, Malaysia; 4 Department of Orthopedic Surgery, Bhumibol Adulyadej Hospital, Bangkok, Thailand; 5 Faculty of Health Sciences and Medicine, Bond University, Queensland, Australia

**Keywords:** Osteoporosis, Biosimilars, Budget impact, Malaysia, Thailand

## Abstract

**Introduction:**

Biologic treatments demonstrate long-term clinical benefits for patients with osteoporosis, but their cost is prohibitive, and access can be limited, particularly in low-income countries. This analysis determined the cost impact of introducing biosimilar denosumab for the treatment of osteoporosis in Malaysia and Thailand.

**Methods:**

A budget impact model was developed to compare the cost of treating female patients aged ≥ 50 years with osteoporosis with biosimilar versus reference denosumab (60 mg/mL every 6 months) from a healthcare payer perspective in Malaysia and Thailand over 5-years. Model inputs included previously published, country-specific prevalence, incidence, mortality, and treatment data, supplemented by expert clinical advice where evidence was limited. Drug acquisition cost was assumed to cost 25% less than the reference product. Further assumptions included no change in osteoporosis incidence and mortality over the time horizon and consistent condition-related costs across both scenarios.

**Results:**

Over 5 years, treatment with the reference denosumab was estimated to cost MYR ~ 864 million (USD ~ 217 million) in Malaysia and THB ~ 179.5 billion (USD ~ 5.2 billion) in Thailand. Biosimilar denosumab was estimated to cost MYR ~ 648 million (USD ~ 162 million) and THB ~ 134.6 billion (USD ~ 3.9 billion), resulting in potential savings of MYR ~ 216 million (USD ~ 54 million) and THB 44.8 billion (USD ~ 1.3 billion), respectively.

**Conclusion:**

The introduction of biosimilar denosumab in Malaysia and Thailand could yield significant cost savings and enhance access to biologic therapies for postmenopausal women with osteoporosis. The inclusion of biosimilar denosumab in national osteoporosis management programs could lead to increased uptake, but it is not explored in this analysis and requires further consideration.

## Introduction

Osteoporosis affects nearly one in five adults globally (18.3% [95% CI: 16.2–20.7]), with higher prevalence rates in women (23.1%) and in developing regions including Malaysia (24.1% in women) [1] and Thailand (20% in women) [2–4]. Between 1990 and 2019, the global burden of deaths linked to low bone mineral density (BMD) dramatically increased, highlighting the escalating burden of fracture-related morbidity and healthcare costs [5]. In Malaysia and Thailand, hip fractures are projected to increase 3.6-fold and 2.8-fold, respectively, by 2050, with annual projected costs of treatment over USD 125 million in Malaysia and over USD 237 million in Thailand [6–8].

Denosumab is a biologic agent used for the treatment of osteoporosis that demonstrated a 68% reduction in the risk of vertebral fractures and a 40% reduction in hip fractures after three years of treatment in a clinical trial (FREEDOM) [9]. In Thailand, denosumab offers a cost-effective osteoporosis treatment option compared with no pharmacologic treatment in postmenopausal women at high risk of fracture [10]. Long-term clinical benefits with denosumab were demonstrated in the corresponding 10-year extension study, including progressive incremental increases in BMD, sustained low rates of vertebral fracture, and further nonvertebral fracture risk without increased risk of adverse events [9]. Despite its clinical benefits, denosumab remains costly, creating financial challenges for healthcare systems, particularly in low-income countries [11–13]. Given the high prevalence of osteoporosis among the aging populations, the economic sustainability of continuous denosumab use is a growing concern [14].

The development of biosimilar denosumab presents an opportunity to reduce treatment costs while maintaining therapeutic effectiveness. Biosimilar denosumab may reduce treatment costs while maintaining comparable therapeutic effectiveness, with potential price reductions of 30%−50% compared with originator biologics [15]. A budget impact analysis (BIA) evaluates the financial implications of introducing new interventions within healthcare budget constraints [16]. It estimates cost savings, informs budget allocation, guides pricing strategies, and supports treatment sustainability [17], hence can aid policymakers to optimize healthcare resources.

In Malaysia, lack of reimbursement and limited resources means that currently less than 8% of eligible patients are receiving the originator denosumab product PROLIA® and less than 10% of eligible patients are receiving XGEVA® [18]. The situation in Thailand is similar, with lack of official reimbursement meaning currently less than 13% of eligible patients receive PROLIA® and less than 7% of eligible patients receive XGEVA® [19].

In Thailand the first denosumab biosimilar received regulatory approval in 2025 following similar launches in Malaysia and India [20]. We performed a budget impact analysis to better understand the impact of introducing biosimilar denosumab for the treatment of osteoporosis from a healthcare payer perspective in Malaysia and Thailand.

## Methods

### Model structure

**Fig. 1:**
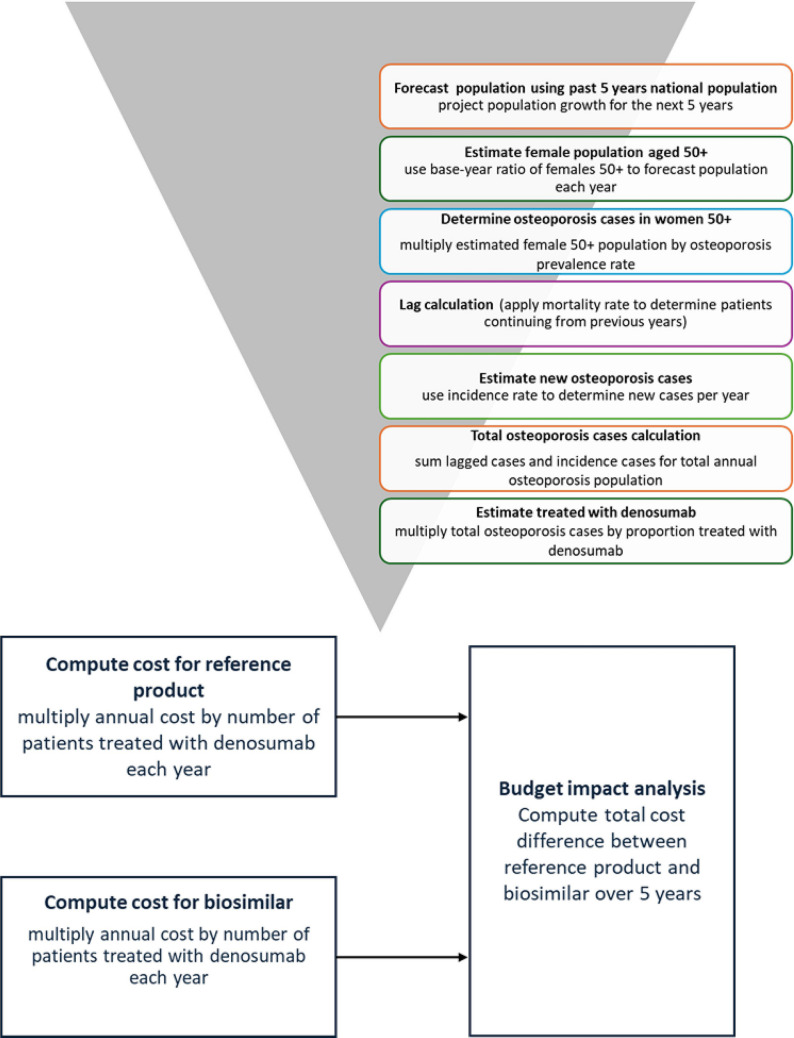
Budget impact model

A budget impact model was developed to compare the cost of treating female patients aged ≥ 50 years with osteoporosis with biosimilar versus reference denosumab from a healthcare payer perspective in Malaysia and Thailand over a 5-year time horizon. The budget impact was calculated as the difference in costs between the two scenarios in each country (Fig. 1). Local currency was converted to USD using conversions rates from Jan 2025 (MYR: 0.25, THB: 0.029).

### Base-case model inputs.

Model inputs and data sources are shown in Table 1. For Malaysia, population-based prevalence and incidence data from a previous budget impact study [21] were combined with Malaysia census data [22] to estimate the proportion of females aged ≥ 50 years in 2024. This proportion was assumed to be constant over time. A linear forecast was undertaken to estimate the country’s total population between 2025 (Year 1) and 2029 (Year 5), by conducting an ordinary least squares regression on population data between 2020 and 2024 and extrapolating to the forecasted years. The proportion of females aged ≥ 50 years was then multiplied by the total population to give the number of females ≥ 50 years old. In Year 1 the prevalent population was assumed to be eligible for treated with denosumab. This was then multiplied by the incidence of osteoporosis in this sub population in each forecast year, to give the total new cases of osteoporosis per year [21]. Of these osteoporosis cases, it was assumed that 16.5% were treated with denosumab [21]. Patients diagnosed each year with osteoporosis were carried through each subsequent year, minus those that were assumed to die according to a crude annual mortality rate amongst females ≥ 50 years of 4.7% [21].

**Table 1:** Model inputs

	Malaysia		Thailand	
	Value	Source	Value	Source
Population inputs				
Population	34,058,800	[22]	71,801,279	[24]
Proportion of females ≥ 50 years of age	0.107	[21]	0.203	[3, 7,25,21 ]
Mortality rate	0.047	[21]	0.034	[25]
Osteoporosis prevalence	16.1%	[21]	27.46%	[26]
Osteoporosis incidence	1.8%	[21]	1.59%	[26]
Proportion treated with denosumab	16.5%	[21]	24.5%	Expert Opinion
Cost inputs (2019 index year)				
Denosumab, annual cost	MYR 1363.4 (MYR 1,487.14 when adjusted for CPI)	[21]	THB 33,212.80	[27]
Biosimilar denosumab, annual cost	MYR 1022.6	Assumption	THB 19,162.50	Assumption

*MYR* Malaysian Ringgit, *THB* Thai Baht

For Thailand, population-based prevalence and incidence data [23] were combined with Thailand World Bank population data [24] to estimate the proportion of female patients aged ≥ 50 years with osteoporosis in 2023. As 2024 population data for Thailand were unavailable at the time of analysis, 2023 data were used as the base year, with five-year forecasting calculated from 2024. The proportion of females aged ≥ 50 years was assumed to be constant over the 5-year period. A linear forecast was applied to estimate the total population of Thailand between 2024 (Year 1) and 2028 (Year 5). Mortality rates were estimated by averaging the age-specific mortality rates across women aged 50 to 85 years from previously published data [3, 7,25,21 ]. A linear forecast was undertaken to estimate the prevalence of osteoporosis in Thailand to 2025 based on data from 2017 to 2022 [26]. There were no incidence data available for osteoporosis, so the incidence data was extrapolated from the same prevalence data [26] (using changes in annual prevalence and adjusting for mortality), the mean of this extrapolated incidence was used in the calculations. It was assumed that the incidence and mortality would remain constant throughout the 5-year time horizon. In Year 1 the prevalent population was assumed to be eligible for treated with denosumab. New patients were introduced into the model annually as incident cases. For Malaysia, the proportion of patients treated with denosumab and biosimilar denosumab was derived from a previous budget impact study [21]. For Thailand, the expected proportion of patients treated with denosumab was not available. In the absence of this data clinical experts from Thailand were asked of these eligible population how many would they expect to accept pharmaceutical therapy (70%) and of those how many would receive denosumab (35%), therefore it was estimated that 24.5% of the eligible population would uptake denosumab. The drug acquisition cost for denosumab was derived from published data [7, 21 ].

The price of denosumab was not available from the Malaysian Ministry of Health Pricing data, so the price was taken from the previously published budget impact analysis [21] and adjusted with CPI to 2025 prices. The price was available for Thailand on the National Drug Policy Division [27]. Entry prices for biosimilars vary between countries but have been reported in studies to typically be 15–35% lower than their respective brand-name reference biologic [28, 29 ]. For this study, the cost of biosimilar denosumab was assumed to be 25% less than that of the reference product. The treatment regimen analyzed was a 60 mg/mL dose of denosumab administered every 6 months. Condition-related costs and drug administration costs were assumed to be consistent across both scenarios and over the 5-year time horizon. All costs were adjusted to 2025 pricing.

### Sensitivity analysis

A one-way sensitivity analysis was conducted by varying the proportion of patients treated by ± 10% for both countries and the cost input of both products by ± 20% to represent the best- and worst-case scenarios. The difference in sensitivity ranges for the proportion of patients treated between countries was due to the greater availability of published country-specific data for Malaysia, whilst the Thai model relied more on assumptions.

## Results

**Fig. 2:**
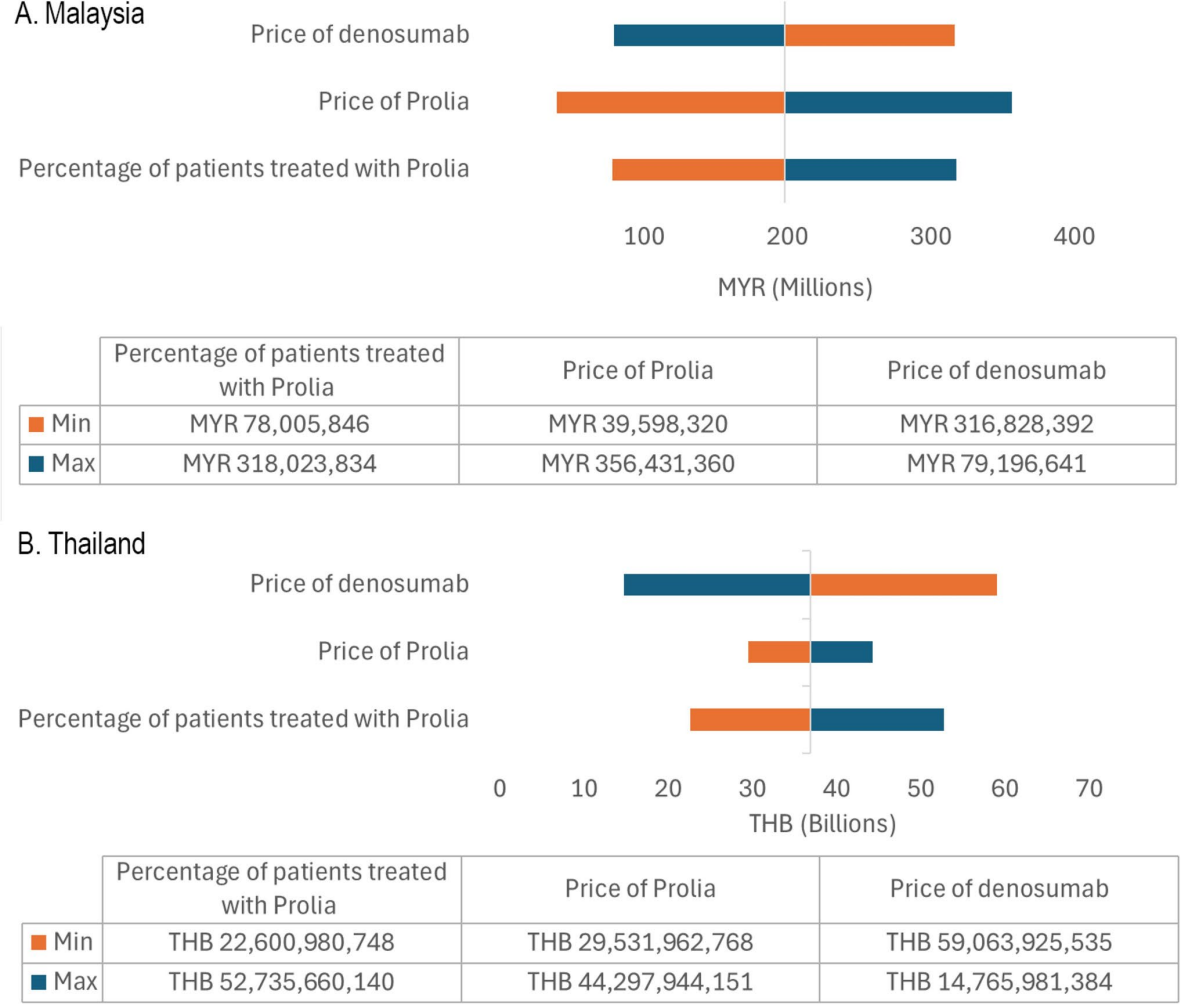
Sensitivity analysis. Max, maximum; min, minimum; MYR, Malaysian Ringgit; THB, Thai Bhat. Base case: MYR 198,014,840 for Malaysia; THB 36,914,953,460 for Thailand

The budget impact of using biosimilar versus reference denosumab in the specified cohort in Malaysia over a time horizon of 5 years is shown in Table 2. The total cost of treating patients with 2 doses of reference product denosumab per year was estimated to range from MYR ~ 154 million (USD ~ 41 million) in 2025 (Year 1) to MYR ~ 191 million (USD ~ 48 million) in 2029 (Year 5), with an overall cost of MYR ~ 864 million (USD ~ 217 million) over 5 years. The cost of treating patients with 2 doses of biosimilar denosumab per year was estimated to range from MYR ~ 116 million (USD ~ 29 million) in 2025 (Year 1) to MYR ~ 143 million (USD ~ 35 million) in 2029 (Year 5,) with an overall cost of MYR ~ 648 million (USD ~ 162 million) over 5 years, equating to a budget impact of MYR ~ −216 million (USD ~ −54 million) compared with denosumab over the 5-year time horizon. In the one-way sensitivity analysis, the budget impact was most sensitive to the price of the reference product in comparison with the price of biosimilar denosumab (budget impact over 5 years: MYR ~ −39.6 to—~ 356.4 million [~ −9.4 to ~ −84.6 million}) and the proportion of patients treated (budget impact over 5 years: MYR ~ −78.0 to ~ −318.0 million[~ −18.5 million to ~ −75.5 million]) (Fig. 2A).

**Table 2:** Total cost and budget impact of using biosimilar denosumab versus denosumab (reference product) for the treatment of osteoporosis in women aged ≥ 50 years in Malaysia

	2024 (Year 0)	2025 (Year 1)	2026 (Year 2)	2027 (Year 3)	2028 (Year 4)	2029 (Year 5)	Overall impact
Females aged ≥ 50 years	3,669,300	3,690,003	3,750,252	3,810,129	3,854,893	3,902,309	
With osteoporosis^a^	590,757	66,420	67,505	68,582	69,388	70,242	
Treated with denosumab	97,475	10,959	11,138	11,316	11,449	11,590	
Treated with denosumab (lag 1)^b^		92,922	10,447	10,618	10,787	10,914	
Treated with denosumab (lag 2)			88,581	9959	10,122	10,284	
Treated with denosumab (lag 3)				84,443	9494	9649	
Treated with denosumab (lag 4)					80,499	9051	
Treated with denosumab (lag 5)						76,738	
Total patients treated	97,475	103,881	110,167	116,337	122,351	128,226	
Price denosumab (MYR)	1,487						
Price biosimilar denosumab (MYR)	1,115						
Annual drug cost (denosumab), MYR		154,485,826	163,833,573	173,009,129	181,953,784	190,690,040	863,972,353 (USD ~ 217 million)
Annual drug cost (biosimilar denosumab), MYR		115,864,370	122,875,180	129,756,847	136,465,338	143,017,530	647,979,265 (USD ~ 162 million)
**Budget impact (-MYR)**		38,621,457	40,958,393	43,252,282	45,488,446	47,672,510	215,993,088 (USD ~ 54 million)

^a^Prevalence rate was used to determine the number of patients with osteoporosis in Year 0. In the subsequent years, incidence rate was used to calculate the number of patients with osteoporosis; ^b^lag provided the patients treated with denosumab from the previous year, accounting for the mortality rate

*MYR* Malaysian Ringgit

The budget impact of using biosimilar versus reference denosumab in the specified cohort in Thailand over a time horizon of 5 years is shown in Table 3. The total cost of treating patients with 2 doses of denosumab per year was estimated to range from THB ~ 34.5 billion (USD ~ 1.0 billion) in 2025 (Year 1) to THB ~ 37.2 billion (USD ~ 1.1 billion) in 2029 (Year 5), with an overall cost of THB ~ 179.5 billion (USD ~ 5.2 billion) over 5 years. The cost of treating patients with 2 doses of biosimilar denosumab per year was estimated to range from THB ~ 25.9 billion (USD ~ 751 million) in 2025 (Year 1) to THB ~ 27.9 billion (USD ~ 810 million) in 2029 (Year 5), with an overall cost of THB ~ 134.6 billion (USD ~ 3.9 billion) over 5 years, equating to a budget impact of THB ~ −44.9 billion (USD ~ −1.3 billion) compared with denosumab over the 5-year time horizon. In the one-way sensitivity analysis, the budget impact was most sensitive to the price of biosimilar compared with reference denosumab (budget impact over 5 years: THB ~ −14 to ~ −59 billion [~—0.48 billion to ~ 1.9 billion]) Fig. 2B. Varying the proportion of patients treated also resulted in a budget impact of THB ~ −22 to ~ −52 billion ([~—0.73 billion to ~ 1.71 billion) over 5 years (Fig. 2B).

**Table 3:** Total cost and budget impact of using biosimilar denosumab versus denosumab (reference product) for the treatment of osteoporosis in women aged ≥ 50 years in Thailand

	2023 (Year 0)	2024 (Year 1)	2025 (Year 2)	2026 (Year 3)	2027 (Year 4)	2028 (Year 5)	Overall impact
Females aged ≥ 50 years	14,600,000	14,628,022	14,648,732	14,671,398	14,695,568	14,719,098	
With osteoporosis^a^	4,009,021	232,986	233,316	233,677	234,062	234,437	
Treated with denosumab	982,210	57,082	57,162	57,251	57,345	57,437	
Treated with denosumab (lag 1)^b^		982,209	55,123	55,201	55,286	55,377	
Treated with denosumab (lag 2)			948,500	53,231	53,306	53,389	
Treated with denosumab (lag 3)				915,948	51,404	51,477	
Treated with denosumab (lag 4)					884,513	49,640	
Treated with denosumab (lag 5)						854,157	
Total patients treated		1,039,291	1,060,785	1,081,630	1,101,854	1,121,476	
Price denosumab^c^ (THB)	25,550						
Price biosimilar denosumab (THB)	19,163						
Annual drug cost (denosumab), THB		34,517,758,643	35,231,646,785	35,923,972,070	36,595,669,505	37,247,364,031	179,516,411,035 (USD ~ 5.2 billion)
Annual drug cost (biosimilar denosumab), THB		25,888,318,983	26,423,735,089	26,942,979,052	27,446,752,129	27,935,523,023	134,637,308,276 (USD ~ 3.9 billion)
**Budget impact (-THB)**		**8,629,439,661**	**8,807,911,696**	**8,980,993,017**	**9,148,917,376**	**9,311,841,008**	**44,879,102,759 (USD ~ 1.3 billion)**

^a^Prevalence rate was used to determine the number of patients with osteoporosis in Year 0. In the subsequent years, incidence rate was used to calculate the number of patients with osteoporosis; ^b^lag provided the patients treated with denosumab from the previous year, accounting for the mortality rate; ^c^price of two units of 60 mg/mL denosumab

*THB* Thai Baht

## Discussion

This study presents a budget impact analysis assessing the potential cost implications of introducing biosimilar denosumab in Malaysia and Thailand. Under the model assumptions, replacing reference denosumab with a lower-cost biosimilar was associated with projected cost savings over a five-year horizon in both settings (Malaysia MYR ~ −198 million (USD ~ −47.0 million); Thailand, THB −35.5 billion (USD ~ −1.12 billion) The potential savings associated with denosumab biosimilars in Malaysia and Thailand are substantial compared with the use of reference denosumab and are driven primarily by the assumed price differential and estimated treatment uptake. Globally the population aged ≥ 60 years is increasing, and with it the prevalence of osteoporosis. Increased life expectancy means the speed of aging among the Asian population is amongst the fastest globally, and with it an increase in the prevalence of osteoporosis [30]. The prevalence of osteoporosis in Thailand is relatively high compared with other Asian countries and, as such, is a major public health concern, warranting the need for lower cost treatment alternatives [2, 3 ]. In Malaysia, the projected increase in osteoporotic fractures to 2050 and associated costs of treatment is the highest amongst countries in Asia [8].

It has already been demonstrated that denosumab is a cost-effective treatment compared with standard of care in Thailand, this was achieved by demonstrating superior efficacy (improving BMD and reducing fracture risk) which translated into better quality of life for women [10]. There is consideration that biosimilars may expand access to biologic therapy for patients, with osteoporosis limited by disposable income, [31] with potential reductions in out-of-pocket expenditure [11]. This is especially important in systems where out of pocket costs can be problematic for low-income earners leading to patients being undertreated [32].

As demonstrated in our analysis, cost savings from biosimilar adoption can lead to a reduction in the financial burden on healthcare systems which enables integration into a value-based health system. Similarly, a study across 57 countries and regions from 2012 to 2019 found that the introduction of biosimilars significantly reduced the cost of biologics globally, demonstrating how increasing biosimilar utilization may help control healthcare costs [33]. Further exploration of biosimilar utilization in the US reported savings from biosimilars of USD 12.4 billion in 2023 [34] and an estimated USD 20.2 billion in 2024 with a cumulative total of USD 56.2 billion since first biosimilar entry in 2015 [35]. Savings have also been demonstrated in Singapore in 2023 after using a value-driven healthcare strategy to implement biosimilars [36]. Given the reduction of costs demonstrated in our analysis it may be possible that biosimilars may contribute to the long-term sustainability of the analyzed healthcare systems and reduce the financial burden of biologic use. As has been demonstrated elsewhere [37]. The use of biosimilars could lead to efficiency gains and reduced financial burden by providing cost-efficiency without compromising efficacy and lowering downstream costs resulting from untreated disease through broader access to treatment [38–40]. Furthermore, they provide stability against market volatility by diversifying biologic supply, which ensures continuity of supply, and reduces the risk of drug shortages [41, 42 ].

The present budget impact analysis has a few limitations. Interpretation of results should consider the simplicity of the model and associated uncertainties. The analysis relies on a limited set of structural assumptions and does not capture potential changes in clinical practice, pricing dynamics, or policy over time. Several of the key inputs in the model were not supported by published data and required softer evidence (such as clinical expert opinion), this was especially the case for Thailand where there was no published data on the uptake of denosumab. In addition, the costs of biosimilars were not available and were based on estimates from other biosimilars in the market. Key inputs such as, and epidemiological parameters, while assuming stable incidence, mortality, and treatment patterns over the time horizon were derived from published sources. Sensitivity analyses demonstrated that results are particularly influenced by uptake and pricing assumptions, highlighting the uncertainty surrounding projected cost savings.. Nonetheless, the sensitivity analysis continued to demonstrate cost saving and the overall analysis provides robust evidence demonstrating the potential cost savings associated with introducing biosimilar denosumab for the treatment of osteoporosis in Malaysia and Thailand.

## Conclusions

This study estimated the short-term budgetary implications of introducing biosimilar denosumab for the treatment of osteoporosis in women aged ≥ 50 years in Malaysia and Thailand. Under the stated assumptions, the use of a lower-cost biosimilar was associated with reduced drug expenditure over a five-year horizon compared with the reference product (Malaysia: MYR ~ 198.0 million (USD ~ 47.0 million) and Thailand: THB 35.5 billion (USD ~ −1.12 billion)). These findings are contingent on assumptions regarding pricing and treatment uptake, including inputs informed by expert opinion, and should be interpreted accordingly. As this analysis focuses solely on financial outcomes, further research incorporating real-world utilisation and clinical outcomes would be required to provide a more comprehensive assessment of impact.

## Data Availability

The data that support the findings of this study are available from the corresponding author upon reasonable request.

## References

[1] Lim PS et al (2005) Bone health in urban midlife Malaysian women: risk factors and prevention. Osteoporos Int 16(12):2069–2079 16234999 10.1007/s00198-005-2003-4

[2] Salari N et al (2021) Global prevalence of osteoporosis among the world older adults: a comprehensive systematic review and meta-analysis. J Orthop Surg Res 16(1):669 34774085 10.1186/s13018-021-02821-8 PMC8590304

[3] Charoenngam N, Pongchaiyakul C (2023) Current issues in evaluation and management of osteoporosis in Thailand. Osteoporos Sarcopenia 9(2):53–59 37496986 10.1016/j.afos.2023.05.002 PMC10366423

[4] Limpaphayom KK et al (2001) Prevalence of osteopenia and osteoporosis in Thai women. Menopause 8(1):65–69 11201518 10.1097/00042192-200101000-00011

[5] Shen Y et al (2022) The global burden of osteoporosis, low bone mass, and its related fracture in 204 countries and territories, 1990–2019. Front Endocrinol (Lausanne) 13:882241 35669691 10.3389/fendo.2022.882241 PMC9165055

[6] Lau EM et al (2001) The incidence of hip fracture in four Asian countries: the Asian Osteoporosis Study (AOS). Osteoporos Int 12(3):239–243 11315243 10.1007/s001980170135

[7] Pongchaiyakul C, Songpattanasilp T, Taechakraichana N (2008) Burden of osteoporosis in Thailand. J Med Assoc Thai 91(2):261–267 18389994

[8] Cheung CL et al (2018) An updated hip fracture projection in Asia: the Asian Federation of Osteoporosis Societies study. Osteoporos Sarcopenia 4(1):16–21 30775536 10.1016/j.afos.2018.03.003 PMC6362950

[9] Kendler DL et al (2022) Denosumab in the treatment of osteoporosis: 10 years later: a narrative review. Adv Ther 39(1):58–74 34762286 10.1007/s12325-021-01936-y PMC8799550

[10] Pongchaiyakul C et al (2020) Cost-effectiveness of denosumab for high-risk postmenopausal women with osteoporosis in Thailand. J Med Econ 23(7):776–785 32063082 10.1080/13696998.2020.1730381

[11] Kvien TK et al (2025) Beyond cost: observations on clinical and patient benefits of biosimilars in real-world settings. BioDrugs 39(4):537–553 40473917 10.1007/s40259-025-00727-z PMC12185555

[12] Assawamakin A et al (2025) Beyond cost: a value-based framework for biosimilar procurement in emerging markets - the Thai experience. J Pharm Policy Pract 18(1):2526827 40656965 10.1080/20523211.2025.2526827 PMC12247093

[13] Shastri K et al (2025) POS0476 Analysing the economic impact of denosumab in the treatment of osteoporosis. Ann Rheum Dis 84(Supplement 1):698–699

[14] Hernlund E et al (2013) Osteoporosis in the European Union: medical management, epidemiology and economic burden. A report prepared in collaboration with the International Osteoporosis Foundation (IOF) and the European Federation of Pharmaceutical Industry Associations (EFPIA). Arch Osteoporos 8(1):136 24113837 10.1007/s11657-013-0136-1 PMC3880487

[15] Moorkens E et al (2017) The market of biopharmaceutical medicines: a snapshot of a diverse industrial landscape. Front Pharmacol 8:314 28642701 10.3389/fphar.2017.00314 PMC5462923

[16] Sullivan SD et al (2014) Budget impact analysis-principles of good practice: report of the ISPOR 2012 Budget Impact Analysis Good Practice II Task Force. Value Health 17(1):5–14 24438712 10.1016/j.jval.2013.08.2291

[17] Garrison LP Jr. et al (2013) Performance-based risk-sharing arrangements-good practices for design, implementation, and evaluation: report of the ISPOR good practices for performance-based risk-sharing arrangements task force. Value Health 16(5):703–19 10.1016/j.jval.2013.04.011 23947963

[18] Abbott (2025) Data on file. Denosumab biosimilar access strategy: Malaysia. Abbott Laboratories

[19] Abbott (2025) Data on file. Denosumab biosimilar access strategy: Thailand. Abbott Laboratories

[20] Abbott. Abbott receives regulatory approval for the first denosumab biosimilar in Thailand, expanding access to bone disease treatment. 2025 September 2025]; Available from: https://www.prnewswire.com/apac/news-releases/abbott-receives-regulatory-approval-for-the-first-denosumab-biosimilar-in-thailand-expanding-access-to-bone-disease-treatment-302538584.html

[21] Choo YW et al (2023) Budget impact of increasing uptake of denosumab for the treatment of postmenopausal osteoporosis in Malaysia. Arch Osteoporos 18(1):145 38030861 10.1007/s11657-023-01358-z

[22] OpenDOSM. Population Table: Malaysia 2025 September 2025]; Available from: https://open.dosm.gov.my/data-catalogue/population_malaysia

[23] Watts JJ, Abimanyi-Ochom J, Sanders KM (2013) Osteoporosis costing all Australians A new burden of diseaseanalysis – 2012 to 2022, in Osteoporosis Australia. Glebe, NSW, Australia. https://healthybonesaustralia.org.au/wp-content/uploads/2022/09/burden-of-disease-analysis-2012-2022.pdf

[24] World Bank Group. World Bank Open Data. 2023 September 2024]; Available from: https://data.worldbank.org/

[25] World Health Organization. Life tables: Life tables by WHO region Global. 2022; Available from: https://apps.who.int/gho/data/view.searo.LIFEREGIONGLOBAL?lang=en

[26] Charoenngam N et al (2024) Trends in osteoporosis prevalence at a tertiary care referral center hospital in Northeastern Thailand: a 20-year analysis (2003–2022). Arch Osteoporos 19(1):64 39042274 10.1007/s11657-024-01425-z

[27] National Drug Policy Division. National Drug Information (2025) [cited 15 August 2025]; Available from https://ndi.fda.moph.go.th/drug_detail/index/?rctype=1C&rcno=6200002®ister=MUMgMi82MihOQik=

[28] Feng K et al (2024) Patient out-of-pocket costs for biologic drugs after biosimilar competition. JAMA Health Forum 5(3):e235429 38551589 10.1001/jamahealthforum.2023.5429 PMC10980968

[29] Pontes MA et al (2023) Comparative price analysis of biological medicines: disparities generated by different pricing policies. Front Pharmacol 14:1256542 38273835 10.3389/fphar.2023.1256542 PMC10808539

[30] Asavamongkolkul A et al (2024) Prevalence of osteoporosis, sarcopenia, and high falls risk in healthy community-dwelling Thai older adults: a nationwide cross-sectional study. JBMR Plus 8(2):ziad020 38505534 10.1093/jbmrpl/ziad020 PMC10945715

[31] Aapro M et al (2025) Biosimilars in osteoporosis treatment: focus on denosumab. Expert Opin Biol Ther 25(8):887–898 40719112 10.1080/14712598.2025.2540471

[32] Mohd Hassan NZA et al (2022) The inequalities and determinants of households’ distress financing on out-off-pocket health expenditure in Malaysia. BMC Public Health 22(1):449 35255884 10.1186/s12889-022-12834-5 PMC8900333

[33] Chen HH, Yemeke T, Ozawa S (2024) Reduction of biologic pricing following biosimilar introduction: analysis across 57 countries and regions, 2012–19. PLoS ONE 19(6):e0304851 38843282 10.1371/journal.pone.0304851 PMC11156405

[34] Biosimilars Council (2025) Biosimilar Medicines Saved $12.4 Billion in 2023. Available online: https://biosimilarscouncil.org/news/biosimilar-medicines-saved-12-4-billion/. Accessed 31 Oct 2025

[35] AAM (2025) U.S. Generic & Biosimilar Medicines Savings Report. Available online: https://accessiblemeds.org/resources/reports/2025-savings-report/. Accessed 31 Oct 2025

[36] Tan SH et al (2024) Impact of Value-Driven Healthcare Strategies for Biosimilar Adoption: The Singapore Story. Pharmacoecon Open 8(5):679–688 39042227 10.1007/s41669-024-00491-w PMC11362392

[37] Association for Accessible Medicines. The U.S. Generic & Biosimilar Medicines Savings Report. 2024 September 2025]; Available from: https://accessiblemeds.org/wp-content/uploads/2025/01/AAM-2024-Generic-Biosimilar-Medicines-Savings-Report.pdf.

[38] Ntais C et al (2024) Fostering healthcare system sustainability through efficient practices: Can adopting biosimilars ease the financial burden of rheumatoid arthritis? F1000Res 13:1128 10.12688/f1000research.156983.2 PMC11983381 40212787

[39] Khraishi M et al (2016) Biosimilars: a multidisciplinary perspective. Clin Ther 38(5):1238–1249 26988243 10.1016/j.clinthera.2016.02.023

[40] Moura CS et al (2015) Early medication use in new-onset rheumatoid arthritis may delay joint replacement: results of a large population-based study. Arthritis Res Ther 17(1):197 26235697 10.1186/s13075-015-0713-3 PMC4522999

[41] Alnaqbi KA et al (2025) Expert consensus recommendations on a biosimilars value framework for the Gulf Cooperation Council countries. Ther Innov Regul Sci 59(1):153–163 39477914 10.1007/s43441-024-00716-4 PMC11706834

[42] Li E et al (2015) Development of biosimilars in an era of oncologic drug shortages. Drug Des Devel Ther 9:3247–3255 26150698 10.2147/DDDT.S75219 PMC4484646

